# Optimising CPAP and oxygen levels to support spontaneous breathing in preterm rabbits

**DOI:** 10.1038/s41390-025-03802-x

**Published:** 2025-01-18

**Authors:** Ebony R. Cannata, Kelly J. Crossley, Erin V. McGillick, Megan J. Wallace, Michelle K. Croughan, Dominic Jurkschat, Sophie J. E. Cramer, Arjan B. te Pas, Stuart B. Hooper, Marcus J. Kitchen

**Affiliations:** 1 The Ritchie Centre, Hudson Institute of Medical Research, Melbourne, VIC Australia; 2 Department of Obstetrics and Gynaecology, Monash University, Melbourne, VIC Australia; 3 School of Physics and Astronomy, Monash University, Melbourne, VIC Australia; 4 Division of Neonatology, Department of Pediatrics, Leiden University Medical Center, Leiden, The Netherlands

## Abstract

**Background:**

Very preterm infants often require respiratory support after birth with current recommendations suggesting the use of continuous positive airway pressure (CPAP) of 4–8 cmH_2_O and an initial fraction of inspired oxygen (FiO_2_) of 0.21–0.3. We have examined the interaction of high and low CPAP and FiO_2_ levels on breathing rates and lung aeration in preterm rabbits.

**Methods:**

Prematurely delivered rabbits (29/32 days gestation) received CPAP of either 5cmH_2_O (5CPAP; *n* = 12) or 15 cmH_2_O (15CPAP; *n* = 14), and a FiO_2_ of either 0.3 (5CPAP/0.3, *n* = 6 or 15CPAP/0.3, *n* = 7) or 0.6 (5CPAP/0.6, *n* = 6 or 15CPAP/0.6, *n* = 7). Breathing rates, lung aeration (functional residual capacity; FRC), lung bulging and air accumulation in the stomach were measured using phase-contrast X-ray imaging.

**Results:**

Kittens receiving 0.6 FiO_2_ had higher breathing rates (5CPAP/0.6: 32.6±6.4 breaths/min; *p* = 0.0064 and 15CPAP/0.6: 36.9±3.5breaths/min; *p* = 0.0010) than 5CPAP/0.3 kittens (11.8±4.1breaths/min). Kittens receiving 15CPAP/0.6 tended to have higher FRC volumes (34.9±4 mL/kg) than kittens receiving 5 cmH_2_O CPAP (5CPAP/0.3: 13.1±6mL/kg; *p* = 0.0675 and 5CPAP/0.6: 13.5±6 mL/kg; *p* = 0.1720) and 15CPAP/0.3 (22.5 ± 6.6 mL/kg; *p* = 0.4245). Lung bulging and air accumulation in the stomach were not different between groups.

**Conclusion:**

Preterm rabbits supported with both 15 cmH_2_O CPAP and 0.6 FiO_2_ increased spontaneous breathing rates and lung aeration without increasing the risk of air in the stomach or lung bulging.

**Impact:**

- While current guidelines recommend the use of low CPAP (4–8 cmH_2_O) and low FiO_2_ levels (0.21–0.3 FiO_2_) to support preterm infants at birth, the optimum levels are unknown.
- This study has shown that 15 cmH_2_O of CPAP and FiO_2_ of 0.6 improved lung aeration and breathing in preterm rabbits, compared with a CPAP of 4 cmH_2_O and FiO_2_ of 0.3.
- These results add to the evidence indicating that initial high CPAP and high FiO_2_ levels, followed by titration of both, enhance respiratory support for preterm newborns.

## Introduction

At birth, very preterm infants have immature, liquid-filled lungs that they struggle to aerate. As a result, they usually require respiratory support, which commonly commences with continuous positive airway pressure (CPAP) delivered non-invasively via a facemask. If the infant is not breathing, or has irregular breathing, they will receive intermittent positive pressure ventilation (IPPV), also via a facemask. However, if the infant’s oxygenation or heart rate remains low, the level of care can escalate leading to endotracheal tube intubation, which bypasses the upper airways to directly ventilate the lungs.^1^ As this type of invasive respiratory support increases the risk of injury and bronchopulmonary dysplasia,^2^ interest has now shifted towards optimising non-invasive strategies to avoid intubation.

Current clinical guidelines suggest using a CPAP of 4–8 cmH_2_O to assist spontaneously breathing preterm infants at birth.^3^ But as very preterm infants have weak inspiratory muscles and immature lungs, higher CPAP levels may be required to assist them to aerate their lungs. Indeed, the application of higher CPAP levels (12–15 cmH_2_O) can better assist the breathing efforts of preterm infants by increasing the hydrostatic pressure gradients needed to move liquid through the airways and across the distal airway wall into lung tissue.^4–6^ However, higher CPAP levels are not currently recommended, due to concerns of lung over-expansion, causing lung injury,^7^ and the risk of air accumulation in the stomach (colloquially known as “CPAP belly”). The latter causes expansion of the abdomen and compression of the lungs,^8,9^ due to rostral displacement of the diaphragm.^10^

Although non-invasive strategies are the preferred mode of respiratory support in the delivery room, when infants are apneic (not breathing) or breathe irregularly, their glottis is mostly closed, which prevents air from entering their lungs.^11,12^ Thus, the ability of non-invasive respiratory approaches to assist lung aeration is largely dependent on the presence of regular breathing, which ensures that the glottis is open. As hypoxia is a potent inhibitor of breathing activity in both the fetus and newborn,^13^ oxygenation status is a critical determinant of breathing activity and, after birth, this is entirely reliant on the onset of pulmonary oxygen exchange. If the lung does not aerate to a sufficient degree, hypoxia worsens, which enhances the suppression of breathing. This leads to apnea and stimulates the glottis to close, which in turn, impedes the application of non-invasive respiratory support that would otherwise increase oxygenation and alleviate the hypoxia.

When the lung is partially aerated at birth, the surface area for gas exchange is limited and so higher fractions of inspired oxygen (FiO_2_) are used to enhance oxygen exchange by increasing the partial pressure gradient for oxygen diffusion. However, current recommendations suggest that the FiO_2_ should initially be low (0.21–0.30) and then titrated based on the infant’s oxygen saturation levels (SpO_2_).^3^ While this recommendation is based on studies showing that prolonged exposure to high oxygen levels causes lung and brain injury, they were mostly conducted in intubated and mechanically ventilated infants.^14^ However, recent studies have questioned these current recommendations in the context of optimizing non-invasive respiratory support and the need to stimulate breathing.^15^ These studies have shown that higher initial FiO_2_ (0.6–1.0) levels increases breathing at birth without necessarily increasing lung aeration (as measured by functional residual capacity; FRC),^16^ and importantly, reduces the burden of neurological injury and mortality.^17^ Furthermore, the most recent meta-analysis has found that commencing resuscitation with a high FiO2 (>0.9) may be associated with reduced mortality (low certainty evidence).^18^

Our aim was to determine the interaction between CPAP and FiO_2_ in optimising lung aeration and the stimulation of breathing, in preterm rabbit kittens at birth. We hypothesised that higher CPAP levels (15 cmH_2_O) would optimise lung aeration, whereas an 0.6 FiO_2_ would provide the greatest stimulation of breathing in the immediate newborn period.

## Materials and methods

### Ethics approval

All animal procedures were approved by the SPring-8 Animal Care and Monash University’s Animal Ethics A Committees. Experiments were conducted in accordance with the National Health and Medical Research Council (NHMRC) Australian code of practice for the care and use of animals for scientific purposes.^19^ Eight pregnant New Zealand White rabbits were used in this study. Methodological reporting is provided per the relevant ARRIVE guidelines.^20^

### Experimental procedures

All experimental procedures were conducted in the experimental hutch, 20B2 at the Japanese synchrotron, SPring-8.

#### Animal preparation and group allocation

At 29/32 days gestational age (~28 weeks human lung development equivalent), pregnant New Zealand White rabbits (*n* = 8) were briefly anesthetised with propofol (intravenous 3 mL; 8 mg/kg bolus) to administer a spinal anaesthesia (2% lignocaine; 0.6 mL and 0.5% bupivacaine; 0.6 mL). Following recovery from propofol, the doe was sedated using intravenous midazolam and butorphanol (0.5 mg/kg/hr and 0.5 mg/kg/hr, respectively, with rate 20 mL/hr) and preterm rabbit kittens were delivered by caesarean section. Preterm rabbit kittens were exposed, one at a time, via a uterine incision before an oesophageal tube was inserted and a custom-made CPAP facemask was fitted to each kitten; the oesophageal tube was passed through a small, sealed hole in the CPAP mask. Kittens were then delivered, weighed and given caffeine (20 mg/kg; intraperitoneally) to stimulate breathing,^21^ naloxone (0.1 mg/kg) to avoid the inhibitory effects of maternally administered butorphanol, and flumazenil (10 μg/kg) to reverse any inhibitory effects of midazolam.

Prior to delivery, all kittens were randomly allocated to one of four treatment groups (*n* = 26), with similar numbers per litter to avoid the risk of litter bias. Each of the treatment groups had different levels of CPAP and FiO_2_ applied during ventilation:

(1) 5 cmH_2_O CPAP + 0.3 FiO_2,_ (5CPAP/0.3; *n* = 6)

(2) 15 cmH_2_O CPAP + 0.3 FiO_2_, (15CPAP/0.3; *n* = 7)

(3) 5 cmH_2_O CPAP + 0.6 FiO_2_, (5CPAP/0.6; *n* = 6)

(4) 15 cmH_2_O CPAP + 0.6 FiO_2_, (15CPAP/0.6; *n* = 7)

Following delivery, kittens were transferred into an imaging hutch and placed on their side on a warmed (38 °C) stage that also contained a mechanical stimulation device. The oesophageal catheter was connected to a pressure transducer to detect breathing efforts (ADInstruments, New South Wales, Australia), and electrocardiogram (ECG) leads were attached to measure heart rate. All physiological data were recorded digitally using a PowerLab and LabChart data acquisition software (ADInstruments, New South Wales, Australia). The initiation of CPAP support was defined as the experiment start (t(0)). All physiological data were recorded continuously throughout the experiment, which was ~12 min in duration. However, X-ray imaging could not commence until ~3 min after initiation of CPAP, allowing time to ensure that the kittens were relatively stable before exiting the imaging hutch. Imaging then continued for a further 7 mins (to ~10 min after commencing CPAP).

#### Respiratory support

The CPAP mask was connected to a custom-made pressure-limited ventilator that applied CPAP at either 5 cmH_2_O or 15 cmH_2_O.^22^ A FiO_2_ of either 0.3 or 0.6 was administered using an oxygen/air blender. If the kittens became apneic and had a low heart rate (<100 beats/min) they received mechanical stimulation. Stimulation commenced using a remote controlled custom-made mechanical stimulation device that was activated from outside the imaging hutch, to avoid disrupting the X-ray imaging.^23^ If apnea persisted, imaging was paused and researchers entered the hutch to perform manual stimulation.

#### Propagation-Based Phase-Contrast X-Ray imaging

Propagation-Based Phase-Contrast X-Ray imaging was used to image lung aeration as previously described.^6,16,24^ Rabbit kittens were placed ~210 m downstream from the source. The X-ray beam was tuned to 24 keV and images were captured by a Hamamatsu ORCA Flash C11440- 22C detector with a tandem lens system, (effective pixel size 15.4 μm, 31.5 (width) × 31.5 (height) mm^2^ field of view) and placed 1.5 m beyond the rabbit kitten. Images were acquired at 10 Hz with an exposure time of 20 ms. After each imaging sequence (~7 min), flat field and dark current images were acquired to correct for variations in X-ray beam intensity and the detector dark current. Lung volumes were calculated from images acquired at FRC and presented as a 60-second trailing average every 30 s from 3 to 10 min after ventilation commenced as previously described in ref. ^24^ At the conclusion of the experiment, images were acquired for flat field and dark field current measurements.

### Physiological analysis

**Fig. 1:**
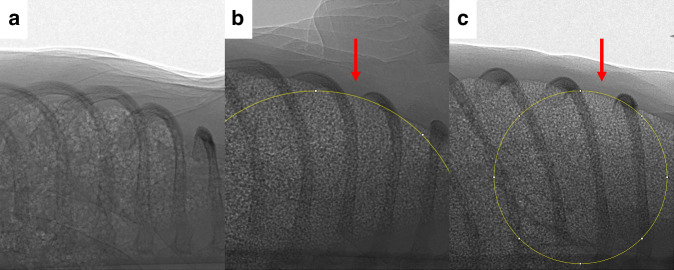
Classifications of lung bulging in rabbit kittens using propagation-based phase-contrast X-ray images. **a** Example of no lung bulging, (**b**) Example of slight lung bulging, (**c**) Example of marked lung bulging. The arrows point to the site of where protrusion of lung tissue between the ribcage was classified (between 4th and 5th rib space). Radius of curvature was calculated by drawing circles (shown in yellow) to fit the edge of the lung. Images have been rotated upright for visual simplicity.

Breathing rates (breaths per minute) were calculated by manually counting breaths detected using the intra-thoracic oesophageal pressure recording and averaged over two minutes. Breathing data reported were collected between 0 and 12 min after ventilation had commenced. All physiological data were corrected for kitten weight, except for heart rate. Heart rate was obtained via an ECG signal obtained in LabChart and averaged every 30 s. Heart rate data reported was between 4.5 and 11.5 min after ventilation had commenced, allowing for heart rate traces to stabilise following the start of the experiment. CPAP belly was assessed from the images and classified according to severity by a single observer (EC) blinded to the pup number and group; the categories were (i) absent, (ii) non-significant, with small amounts of air not impacting diaphragmatic movement and (iii) significant, with the presence of air impeding and altering the shape of the diaphragm. Lung bulging was assessed using Image J,^25^ and quantified by measuring the radius of curvature of lung tissue protruding between the ribs (between the 4th and 5th ribs; Fig. 1) as previously described.^6^

### Statistical analysis

Data were analysed using GraphPad Prism Version 9.2 (GraphPad Software, La Jolla California). Statistical significance was accepted as *p* ≤ 0.05. A power analysis was calculated using G*Power Version 3.1.9.6 to obtain the sample size, with an estimated effect size of *d* = 2.5 and a statistical power of 0.8; giving a *n* = 7.The data were tested for normality using a Shapiro-Wilk test and where possible transformed if not normally distributed; breathing rate data were transformed using a square root. Normally distributed data with two or more independent groups were analysed using a one-way ANOVA. Parametric data with two or more independent groups and measured over time were analysed using a mixed-effect model analysis. An area under the curve (AUC) analysis was used to compare differences between breathing rates and FRC changes over time between groups. A one-way ANOVA with a Tukey’s multiple/column comparison was applied to determine significance levels. Non-parametric data that could not be transformed with two or more independent groups were analysed using a Kruskal Wallis analysis. If the data were parametric and statistically significant, then a Holm-Šídák post-hoc test was used and if the data were non-parametric and statistically significant, a Dunn’s post-hoc test was used. Categorical data was analysed using a Fisher’s exact test.

## Results

### Pre-experimental animal characteristics

Kitten weights were not different between groups, (*p* = 0.9038); Table 1.

**Table 1:** Summary Statistics.

	5CPAP/0.3, (*n* = 6)	15CPAP/0.3, (*n* = 7)	5CPAP/0.6, (*n* = 6)	15CPAP/0.6, (*n* = 7)	*p*-value
Age, (GA)	29	29	29	29	-
Weight, (g)	31.0 ± 2.1	32.1 ± 0.9	32.5 ± 3.2	33.3 ± 2.2	0.9038
Average BR, (breaths/min)	11.79 ± 4.1 *a*	20.8 ± 4.3 *b*	32.6 ± 6.4 *b*	36.9 ± 3.5 *b*	0.0009
Maximum BR, (breaths/min)	29.67 ± 3.8 *a*	51.7 ± 5.6 *ac*	58.0 ± 7.2 *bc*	63.1 ± 3.2 *bc*	0.0010
Average FRC, (mL/kg)	13.1 ± 6.5	22.5 ± 6.6	17.1 ± 6.0	34.9 ± 4.1	0.0726
Manual Stimulation Throughout Ventilation, *n* (%)	1/6 (16.7%)	0/7 (0%)	0/6 (0%)	0/7 (0%)	0.4615
Average Mechanical Stimulation, (*n*)	4.3 ± 1.1	3.9 ± 0.7	2.3 ± 1.1	1.3 ± 0.6	0.0728
Average HR, (beats/min)	141 ± 15	147 ± 15	181 ± 18	190 ± 8 #	0.0716
CPAP Belly, non-significant:significant (*n*)	3:0	3:1	4:1	2:2	0.7700
Lung Bulging, slight:marked (*n*)	1:0	3:0	1:1	4:2	0.1661

Data is presented as *n* (%) or a ratio for categorical data and mean ± SEM for normal parametric data. The values in the average mechanical stimulation row are the average occurrence of stimulation events for each kitten in that group. #exclude *n* =1 due to poor trace. *GA* gestational age, *BR* breathing rate, *FRC* functional residual capacity, *CPAP* continuous positive airway pressure. *a, b, c*… = column comparison symbols where different letters are significantly different.

### Breathing

**Fig. 2:**
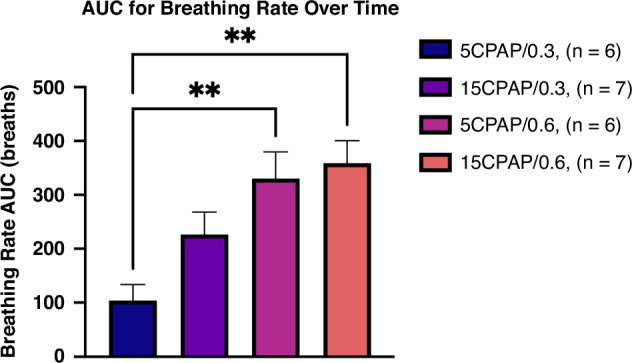
AUC for breathing rate changes over time. Number of breaths taken between 2 and 12 min after birth between different applications of continuous positive airway pressure (CPAP) and fraction of inspired oxygen (FiO_2_). Data is presented as area under the curve (AUC) ± SEM. **p < 0.01.

**Fig. 3:**
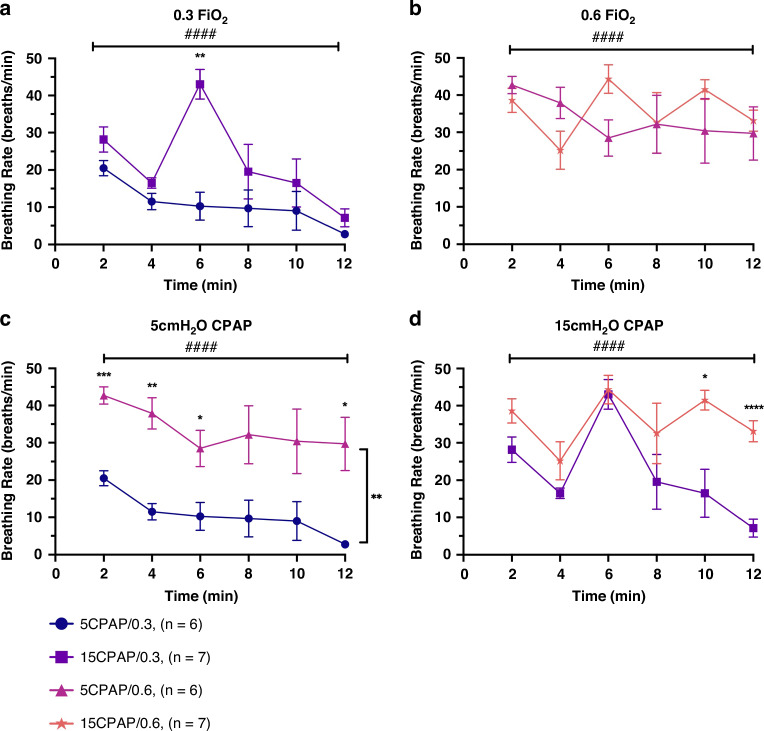
Breathing rate. **a** Effect of CPAP on breathing rate over time in kittens receiving a FiO_2_ of 0.3, (5CPAP/0.3 vs. 15CPAP/0.3), **b** Effect of CPAP on breathing rate over time in kittens receiving a FiO_2_ of 0.6 (5CPAP/0.6 vs. 15CPAP/0.6), **c** Effect of FiO_2_ on breathing rate over time in kittens receiving 5 cmH_2_O CPAP, (5CPAP/0.3 vs. 5CPAP/0.6), **d** Effect of FiO_2_ on breathing rate over time in kittens receiving 15 cmH_2_O CPAP, (15CPAP/0.3 vs. 15CPAP/0.6). Data is presented as mean ± SEM. *p < 0.05, **p < 0.01, ***p < 0.001, ****p < 0.0001 (between groups). ####p < 0.0001 (over time). CPAP continuous positive airway pressure (cmH_2_O). FiO_2_ fraction of inspired oxygen (%).

Breathing rates were quite variable within each pup and within groups over time. The average breathing rate was significantly lower in the 5CPAP/0.3 group (11.8 ± 4.1 breaths/min) compared with both the 5CPAP/0.6 (32.6 ± 6.4 breaths/min; *p* = 0.0064) and 15CPAP/0.6 (36.9 ± 3.5 breaths/min; *p* = 0.0010) groups and tended to be lower than the 15CPAP/0.3 (20.8 ± 4.3 breaths/min; *p* = 0.2112); Table 1. When assessed by measuring the area under the curve for breathing rate between 0 and 12 min after birth, the total number of breaths increased from 104.1 ± 29.5 breaths in the 5CPAP/0.3 group to 226.5 ± 41.5 breaths in the 15CPAP/0.3 group to 330.3 ± 49.6 breaths in the 5CPAP/0.6 group and to 358.9 ± 41.5 breaths in the 15CPAP/0.6 group, (*p* = 0.0012; Fig. 2). A mixed-model analysis of the breathing rate over time data showed statistically significant effects of time (*p* < 0.0001) and treatment (*p* < 0.0001); Fig. 3.

#### Effect of CPAP on breathing rates

In kittens receiving a FiO_2_ of 0.3, 15 cmH_2_O of CPAP significantly increased breathing rates, compared with a CPAP of 5 cmH_2_O, (15CPAP/0.3 vs 5CPAP/0.3; *p* = 0.0032) at 6 minutes after birth. However, breathing rates then rapidly declined in the 15CPAP/0.3 group and were similar to the 5CPAP/0.3 group at 12 min (Fig. 3a). In kittens receiving a FiO_2_ of 0.6, CPAP level had no significant effect on breathing rates and were similar in the 5CPAP/0.6 and 15CPAP/0.6 groups over time (Fig. 3b).

#### Effect of oxygen on breathing rates

In kittens receiving CPAP levels of 5 cmH_2_O, a FiO_2_ of 0.6 (5CPAP/0.6) significantly increased breathing rates at 2 min (*p* = 0.0002), 4 min (*p* = 0.0027), 6 min (*p* = 0.0443), and 12 min (*p* = 0.0387), compared with a FiO_2_ of 0.3 (5CPAP/0.3; Fig. 3c). In kittens receiving CPAP levels of 15 cmH_2_O, breathing rates were initially similar in kittens receiving a FiO_2_ of 0.3 (15CPAP/0.3) and 0.6 (15CPAP/0.6). However, breathing rates were significantly reduced in the 0.3 FiO_2_ group (15CPAP/0.3) compared with the 0.6 FiO_2_ group (15CPAP/0.6); by 10 min (*p* = 0.0467) and 12 min (*p* < 0.0001) (Fig. 3d).

#### Maximum breathing rates

Maximum breathing rates between 0 and 12 minutes after birth were significantly lower in the 5CPAP/0.3 group (29.67 ± 3.8 breaths/min) compared with all other groups; 15CPAP/0.3 (51.7 ± 5.6 breaths/min; *p* = 0.0250), 5CPAP/0.6 (58.0 ± 7.2 breaths/min; *p* = 0.0057) and 15CPAP/0.6 (63.1 ± 3.2 breaths/min; *p* = 0.0009; Table 1).

### Functional residual capacity

**Fig. 4:**
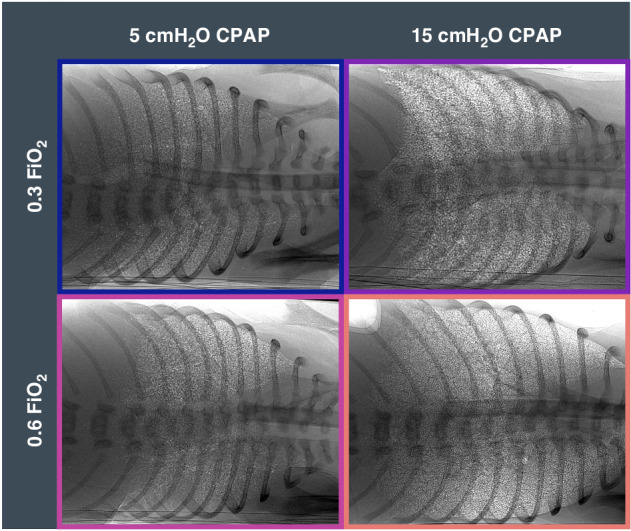
Average functional residual capacity. Phase contrast X-ray images showing the average functional residual capacity (FRC) that was recorded for each group. Kittens received continuous positive airway pressures (CPAP) of either 5 cmH_2_O (left) or 15 cmH_2_O (right), or a fraction of inspired oxygen (FiO_2_) of 0.3 (top) or 0.6 (bottom). Kittens are oriented on their sides during imaging. The bordered colours match graph colours.

**Fig. 5:**
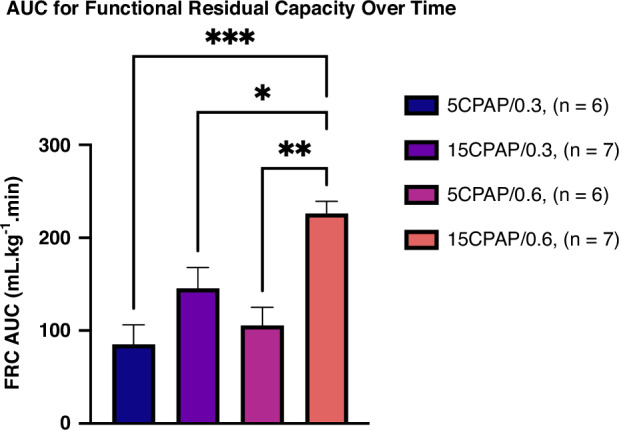
AUC for the increase in functional residual capacity (FRC) over time. The FRC.time integral (mL.kg^−1^.min) calculated between 3 and 10 min after ventilation commencement in kittens receiving either a continuous positive airway pressure (CPAP) of 5 or 15 cmH_2_O or a fraction of inspired oxygen (FiO_2_) of either 0.3 or 0.6. *p < 0.05. **p < 0.01. ***p < 0.001. Data is presented as area under the curve (AUC) ± SEM.

Although average FRC levels were not different between treatment groups, kittens receiving 15 cmH_2_O CPAP with 0.6 FiO_2_ tended to have higher FRC volumes (34.8±4.1 mL/kg) than kittens receiving 5 cmH_2_O CPAP (0.3 FiO_2_: 13.1±6 mL/kg; *p*=0.0675 and 0.6 FiO_2_: 17.1±6 mL/kg; *p* = 0.1720) and, 15 cmH_2_O CPAP with 0.3 (22.5 ± 6.6 mL/kg; *p* = 0.4245); Table 1. Representative X-ray images of the average FRC for each group are shown; Fig. 4. When FRC volumes were assessed by measuring the volume accumulated over a fixed time (area under the curve between 3 to 10 min after birth), total FRC volumes in the 15CPAP/0.6 group (226 ± 13 mL.kg^-1^.min) were significantly higher than the 5CPAP/0.3 (85 ± 21 mL.kg^-1^.min; *p*=0.0002), 5CPAP/0.6 (106 ± 19 mL.kg^-1^.min; *p* = 0.0012) and 15CPAP/0.3 (146 ± 23 mL.kg^-1^.min; *p* = 0.0270) groups (Fig. 5).

#### Effect of CPAP on FRC Levels

**Fig. 6:**
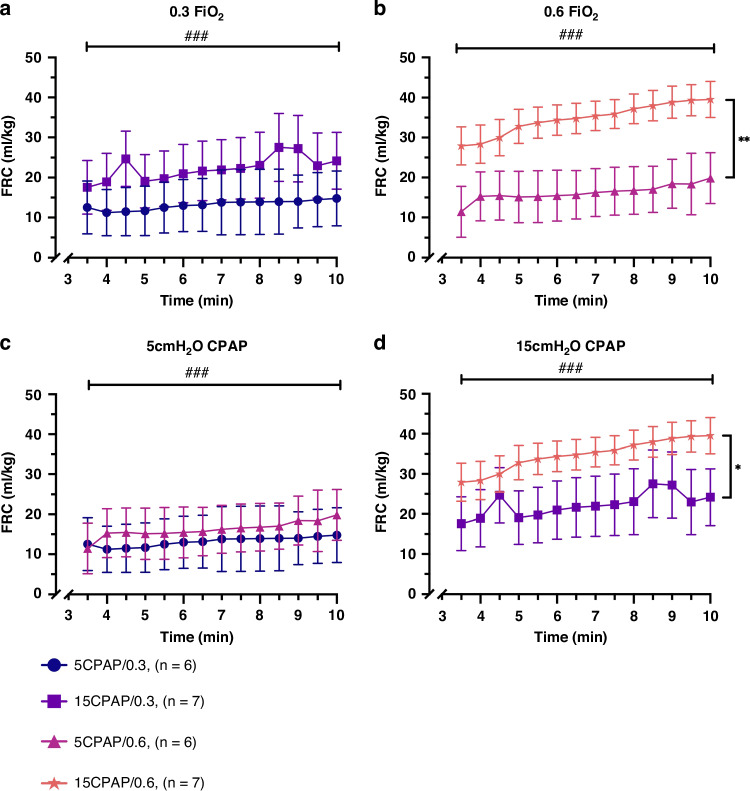
Functional residual capacity (FRC). **a** Effect of CPAP on FRC over time in kittens receiving a FiO_2_ of 0.3, (5CPAP/0.3 vs. 15CPAP/0.3). **b** Effect of CPAP on FRC over time in kittens receiving a FiO_2_ of 0.6 FiO_2_ (5CPAP/0.6 vs. 15CPAP/0.6). **c** Effect of FiO_2_ on FRC over time in kittens receiving 5 cmH_2_O CPAP, (5CPAP/0.3 vs. 5CPAP/0.6). **d** Effect of FiO_2_ on FRC over time in kittens receiving 15 cmH_2_O CPAP, (15CPAP/0.3 vs. 15CPAP/0.6). Data is presented as mean ± SEM. ###*p* < 0.001 (over time). *p < 0.05. **p < 0.01 (between groups). FRC = functional residual capacity (mL). CPAP continuous positive airway pressure (cmH_2_O). FiO_2_ fraction of inspired oxygen (%).

When given a FiO_2_ of 0.3, kittens receiving a CPAP of 15 cmH_2_O (15CPAP/0.3) tended to have a higher FRC levels than kittens receiving a CPAP of 5 cmH_2_O (5CPAP/0.3), although this difference was not quite significant (*p* = 0.1509; Fig. 6a). However, when given a FiO_2_ of 0.6, kittens receiving a CPAP of 15 cmH_2_O (15CPAP/0.6) had significantly higher FRC levels than kittens receiving a CPAP of 5 cmH_2_O (5CPAP/0.6; *p* = 0.0012); Fig. 6b.

#### Effect of FiO_2_ on FRC levels

When kittens were given a CPAP of 5 cmH_2_O, kittens receiving a FiO_2_ of 0.3 had similar FRC levels as kittens receiving a FiO_2_ of 0.6 (*p* = 0.8842); Fig. 6c. However, when kittens were given a CPAP of 15 cmH_2_O, kittens receiving a FiO_2_ of 0.6 had significantly higher FRC levels than kittens receiving a FiO_2_ of 0.3 (*p* = 0.0270; Fig. 6d).

### Secondary outcomes; stimulation, heart rate, CPAP belly and lung bulging

#### Stimulation

The frequency that the kittens received mechanical stimulation (using a mechanical stimulator activated remotely) was not significantly different between groups (*p*=0.0728). However, kittens receiving an 0.6 FiO_2_ tended to receive less mechanical stimulation on average compared to kittens that received 0.3 FiO_2_; Table 1. Only one kitten, which was in the 5CPAP/0.3 group, required manual stimulation during the experimental period (*p* = 0.4615); Table 1.

#### Heart rate

Assessed over time (area under the curve), kittens in the 5CPAP/0.3 group (912±48 beats) had a significantly lower heart rate over time than kittens in the 5CPAP/0.6 (1166±61 beats; *p*=0.0230) and 15CPAP/0.6 (997±61 beats; *p*=0.0337) groups (Supplementary Fig. 1). Kittens in the 15CPAP/0.6 (1228±49 beats) also had a significantly higher heart rate over time than kittens in the 15CPAP/0.3 (*p*=0.0337) group. While average heart rates at specific time points were not significantly different between groups, average heart rates tended to be greater in kittens that received 0.6 FiO_2_. In kittens receiving a FiO_2_ of 0.3, average heart rates were 141 ± 15 (5CPAP/0.3) and 147 ± 15 beats/minute (15CPAP/0.3) whereas in kittens receiving a FiO_2_ of 0.6, had average heart rates of 181 ± 18 (5CPAP/0.6) and 190 ± 8 beats/min (15CPAP/0.6; *p*=0.0716); Table 1.

#### Air accumulation in the stomach; “CPAP Belly”

The severity of CPAP belly was not statistically different between groups, (*p*=0.7700). If air did enter the stomach, the observed timing of onset was not consistent between kittens, however, the mean onset time of CPAP belly was 6.1 mins after starting the experiment. In all kittens, 46.2% had non-significant levels of air accumulation and 15.4% had significant amounts of air. Out of the four kittens that had a severe incidence of CPAP belly, they had received either 15 cmH_2_O CPAP (3/4 (75%)) or 0.6 FiO_2_ (3/4 (75%)); Table 1.

#### Lung bulging

Although not significant, the incidence of lung bulging at a maximum inspiration tended to be highest in the 15CPAP/0.6 group (slight bulging, *n* = 4; marked bulging, *n* = 2), followed by 15CPAP/0.3 (slight bulging, *n* = 3; marked bulging, *n* = 0), 5CPAP/0.6 (slight bulging, *n* = 1; marked bulging, *n* = 1), and the 5CPAP/0.3 group (slight bulging, *n* = 1; marked bulging, *n* = 0), (*p* = 0.1661); Table 1.

## Discussion

In this study, we explored the inter-relationship between different CPAP and FiO_2_ levels on breathing rates and lung aeration in preterm rabbit kittens. We found that higher FiO_2_ levels (0.6) enhanced breathing whereas higher CPAP levels (15 cmH_2_O) enhanced lung aeration, with both acting largely independently of each other. As a result, kittens receiving 15 cmH_2_O of CPAP and a FiO_2_ of 0.6 had higher FRCs and breathing rates, tended to have higher heart rates, and tended to require less stimulation than kittens receiving lower CPAP and FiO_2_ levels. Indeed, kittens that received 5 cmH_2_O of CPAP and a FiO_2_ of 0.3 had the worst outcomes. These included, reduced breathing rates, lower FRC levels, the need for additional stimulation, and lower heart rates. As current clinical guidelines recommend that, at birth, non-invasive respiratory support for very preterm infants should commence with a CPAP of 5–8 cmH_2_O and a FiO_2_ of 0.3 (or less), our results indicate that this recommendation may need re-evaluation.

### Development of FRC

Lung aeration after birth primarily results from hydrostatic pressure gradients generated by either inspiratory efforts or positive pressure inflations provided by a ventilator.^26–28^ These pressure gradients drive the movement of liquid distally through the airways and then across the distal (alveolar) airway wall into the surrounding lung tissue.^29^ As high CPAP levels act to increase the pressure gradient generated by inspiration, it is not surprising that a CPAP of 15 cmH_2_O was substantially better than 5 cmH_2_O for enhancing lung aeration and generating an FRC. However, it is interesting that simply increasing breathing activity, by increasing FiO_2_ levels, is by itself, not sufficient to significantly enhance lung aeration. Indeed, FRC levels in the 5CPAP/0.3 and 5CPAP/0.6 groups were similar despite markedly different breathing rates. While surprising, it is consistent with the finding that mechanically ventilated preterm rabbits require a positive end-expiratory pressure (PEEP) to accumulate an FRC.^27^ However, this requirement for PEEP was thought to primarily result from tracheal intubation, which by-passes the glottis and negates its capacity to restrict expiratory gas flow and retain air in the lung at end-expiration.^27^ Our finding that FRC levels were similar in both 5CPAP groups, despite very different breathing rates, indicates that even spontaneously breathing in very preterm neonates with an intact glottis require positive airway pressures to retain air in their lungs at end-expiration.

We used large differences in both CPAP (5 vs 15 cmH_2_O) and FiO_2_ (0.3 vs 0.6) to identify any independent and contributory effects of CPAP and FiO_2_ may have on breathing and lung aeration in preterm kittens. In kittens receiving a CPAP of 15 cmH_2_O, FRC levels tended (*p*=0.056) to be higher when combined with a FiO_2_ of 0.6 (34.8 ± 4.1 mL/kg) compared to a FiO_2_ of 0.3 (23.2 ± 5.7 mL/kg). Although it is unclear why FRC levels would be higher in 15CPAP/0.6 kittens, it is possible that lower breathing rates in 15CPAP/0.3 kittens contributed. However, we have previously reported FRCs of ~20 mL/kg in preterm kittens receiving a CPAP of 15 cmH_2_O and a FiO_2_ of 1.0 with similar breathing rates as those measured in our 15CPAP/0.6 kittens.^6^ While no kittens in this study experienced a pneumothorax and rates of lung bulging and CPAP belly were similar between groups, we would normally consider FRC levels of 35 mL/kg to be higher than desired (normally 15–30 mL/kg).^28^ Indeed, as high end-expiratory pressures are associated with an increase in pulmonary vascular resistance,^30^ it is possible that pulmonary blood flow would be reduced in these kittens. However, we have previously shown in lambs that high CPAP levels do not impede the increase in pulmonary blood flow at birth, likely because the maximum pressure transmitted into the lower airways was limited to 12 cmH_2_O despite the application 15 cmH_2_O at the mouth opening.^31^

The objective of high CPAP levels is to assist preterm newborns generate the transpulmonary pressures required to move lung liquid distally through the airways and across the distal airway wall.^29^ However, after the lungs have aerated, it is unlikely that high CPAP levels will continue to be advantageous and so could be reduced.^6^ This suggestion is consistent with the concept that the purpose of CPAP immediately after birth is multi-dimensional and changes as the lung transitions. That is, the initial objective is to assist in airway liquid clearance, but then changes into helping prevent airway liquid re-entry and distal airway collapse at end-expiration.^29^ As this requires less pressure, the pressure could be reduced, but it is unclear how quickly this should occur and to what level should the pressure be reduced. We have recently shown that reducing CPAP levels from 15 to 5 cmH_2_O within the first 10 min of birth led to a loss of FRC, whereas a reduction from 15 to 8 cmH_2_O did not in preterm rabbit kittens.^6^ These findings indicate that, during the transition from a liquid filled to air filled lung, a CPAP of 5 cmH_2_O is too low, whereas 8 cmH_2_O or above is more appropriate. However, it is likely that CPAP levels can be reduced further as liquid is cleared from lung tissue and the lung becomes less “oedematous”, but this may take a few hours,^32^ as the lung completes its transition into a gas exchange organ.^29^ A feasibility trial in preterm human infants also showed a quicker stabilisation of heart rates and reduced mask ventilation times with downwards titration of CPAP from 15 to 8 cmH_2_O.^33^ This supports the idea that CPAP levels should be titrated to match the aerated state of the lung during airway liquid clearance after birth.

### Effect of FiO_2_ on breathing

Irrespective of CPAP level, kittens receiving a FiO_2_ of 0.6 had higher breathing rates than kittens receiving a FiO_2_ of 0.3, which supports the concept that oxygenation is a major determinant of breathing in the newborn. The breathing rates we measured (30–40 breaths/min) are similar to those we have recently measured in preterm rabbit kittens of similar gestational age.^6,16^ Both before and after birth, hypoxia is a potent inhibitor of breathing, which is detected by peripheral chemoreceptors that signal into the respiratory centre via specific nuclei located in the upper lateral pons that inhibit breathing.^34^ As a result, even mild hypoxia in the newborn inhibits breathing, which in turn causes the glottis to close and prevents air from entering the lung.^11^ This rationale explains the recent findings that higher FiO_2_ levels (0.6–1.0) results in higher breathing activity in preterm infants, lambs and rabbit kittens.^4,16,35,36^ For example, in preterm infants, an initial FiO_2_ of 1.0, followed by downwards titration as required, resulted in quicker acquisition of peripheral oxygen saturation (SpO_2_) targets, fewer and shorter periods of hypoxia, higher minute volumes and a tendency for a reduced overall oxygen exposure than infants commencing in low oxygen levels that were then titrated up.^4,36^ Thus, it is not surprising that a recent meta-analysis has shown that the high initial FiO_2_ levels may be associated with reduced mortality rates in very preterm infants.^18^

As higher CPAP levels improve lung aeration, we expected that the combination of high CPAP and high inspired oxygen levels would result in the highest breathing rates. This is because higher levels of lung aeration increase the surface aera for gas exchange, which increases oxygen exchange efficiency. Thus, the combined effect of a higher oxygen exchange efficiency and a higher oxygen gradient for diffusion must lead to higher oxygen levels in the newborn. However, as breathing rates were similar in the 15CPAP/0.6 and 5CPAP/0.6 groups, despite large differences in FRC, the relationship between oxygenation levels and breathing rates is multi-dimensional. That is, while a lack of oxygen (hypoxia) is a potent inhibitor of breathing, oxygen per se is not a stimulus for breathing, but primarily acts by preventing hypoxia. As such, when oxygenation increases above a “hypoxic threshold”, increasing the fraction of inspired oxygen further does not further increase breathing rates and may become inhibitory.^37^ Thus, even though oxygen exchange capacity was reduced in the 5CPAP/0.6 kittens, indicated by lower lung aeration levels, a FiO_2_ of 0.6 was likely sufficient to maintain oxygenation levels above the hypoxic threshold, despite lower levels of lung aeration. It is possible, therefore, that FiO_2_ levels of 0.6 were higher than needed, whereas FiO_2_ levels of 0.3 were too low. Indeed, kittens in the 15CPAP/0.3 group had lower breathing rates and tended to have lower heart rates and required almost twice the amount of stimulation as 15CPAP/0.6 kittens. While the optimum FiO_2_ level must initially change (decrease) as lung aeration increases, the threshold at which higher oxygenation levels have no additional stimulatory effect on breathing is yet to be determined.

All preterm kittens received caffeine as a respiratory stimulant in this study, which acts by blocking the inhibitory neurotransmitter adenosine within the respiratory centre of the brainstem.^38,39^ However, the effect of the administered caffeine may have differed in different groups, particularly as hypoxia is known to stimulate increased adenosine release which may reduce the activity of caffeine on adenosine receptors in the brainstem.

### Limitations

Although preterm rabbit kittens are a good model for studying respiratory function in preterm infants and are an ideal size to image breath-by-breath changes in lung aeration, they are too small to measure arterial oxygen levels. As a result, we were unable to directly relate oxygenation levels and breathing activity to determine how hypoxic or hyperoxic these kittens may have become. Nevertheless, as SpO_2_ monitoring is relatively common in the delivery room and FiO_2_ levels are mostly titrated to achieve oxygen saturation levels within target ranges, these adverse events are less likely to occur in preterm infants.

While we found that high CPAP levels did not increase the risk of lung over expansion or CPAP belly and no pneumothoraces were observed, this study was not powered to detect these adverse events. As such, the safe use of high CPAP levels beyond the current guidelines are yet to be determined.

On the other hand, while the primary outcomes of this study (breathing rates and lung aeration) are totally reliant on the presence of spontaneous breathing, it was necessary to apply external mechanical stimuli to prevent the onset of apnea. As this stimulation increases spontaneous breathing activity and as different groups tended to receive different levels of stimulation, it is highly likely that this treatment reduced the true differences in breathing rates between groups.

## Conclusions

We have shown that the combined use of high (15 cmH_2_O) CPAP and high (0.6) FiO_2_ levels significantly increase both lung aeration levels and breathing rates compared to low (5 cmH_2_O) CPAP and low (0.3) FiO_2_ levels. However, the effects of CPAP and FiO_2_ level appeared to work largely independently of each other with FiO_2_ having the greatest effect on breathing rate and CPAP having the greatest effect on lung aeration. While current resuscitation guidelines recommend the use of low FiO_2_ levels (< 0.3) and CPAP levels (5–8 cmH_2_O), preterm rabbit kittens receiving these parameters had the worst outcomes in terms of breathing rates, lung aeration levels, heart rates and need for mechanical stimulation. It is possible that, like FiO_2_ levels, CPAP levels in the delivery room need to be titrated according to need, starting high and then decreasing as the lung aerates and the gas exchange potential increases.

## Supplementary information

Supplementary Figure

## Data Availability

Data will be made available upon reasonable request.
