# Supplementary material for: Optimising CPAP and oxygen levels to support spontaneous breathing in preterm rabbits
Source: Pediatr Res. 2025 Jan 18;99(4):1583–90. doi: 10.1038/s41390-025-03802-x (PMC13102708; doi:10.1038/s41390-025-03802-x)
Supplement: Supplementary file 1 — Supplementary Figure [file 41390_2025_3802_MOESM1_ESM.pdf]

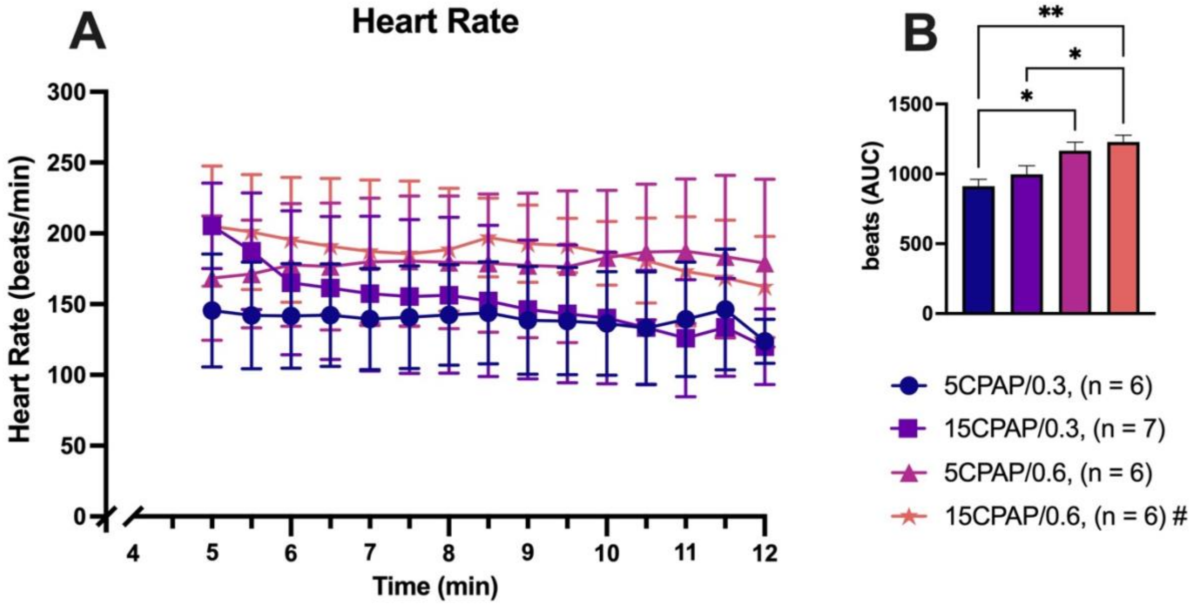

Supplementary Figure 1. ***Heart rate.*** **A)** Mean (± SEM) heart rate values measured over the experimental period. **B)** Total number of heart beats (measured from the area under the curve; AUC) for the experimental period (4.5 to 11.5 mins). CPAP = continuous positive airway pressure (cmH_2O). #exclude n =1 due to poor trace.
